# Circulating Angiotensin-(1–7) Is Reduced in Alzheimer’s Disease Patients and Correlates With White Matter Abnormalities: Results From a Pilot Study

**DOI:** 10.3389/fnins.2021.636754

**Published:** 2021-04-06

**Authors:** Victor Teatini Ribeiro, Thiago Macedo e Cordeiro, Roberta da Silva Filha, Lucas Giandoni Perez, Paulo Caramelli, Antônio Lúcio Teixeira, Leonardo Cruz de Souza, Ana Cristina Simões e Silva

**Affiliations:** ^1^Laboratório Interdisciplinar de Investigação Médica, Faculdade de Medicina, Universidade Federal de Minas Gerais (UFMG), Belo Horizonte, Brazil; ^2^Departamento de Clínica Médica, Faculdade de Medicina, Universidade Federal de Minas Gerais (UFMG), Belo Horizonte, Brazil; ^3^Neuropsychiatry Program and Immuno-Psychiatry Lab, Department of Psychiatry and Behavioral Sciences, University of Texas Health Science Center at Houston, Houston, TX, United States

**Keywords:** Alzheimer’s disease, renin angiotensin system, Angiotensin-(1–7), angiotensin II, white matter hypointensities, cerebrovascular lesions

## Abstract

**Introduction:**

Alzheimer’s disease (AD) is the leading cause of dementia worldwide. Despite the extensive research, its pathophysiology remains largely unelucidated. Currently, more attention is being given to the disease’s vascular and inflammatory aspects. In this context, the renin-angiotensin system (RAS) emerges as a credible player in AD pathogenesis. The RAS has multiple physiological functions, conducted by its two opposing axes: the classical, led by Angiotensin II (Ang II), and the alternative, driven by Angiotensin-(1–7) [Ang-(1–7)]. These peptides were shown to interact with AD pathology in animal studies, but evidence from humans is scarce. Only 20 studies dosed RAS molecules in AD patients’ bloodstream, none of which assessed both axes simultaneously. Therefore, we conducted a cross-sectional, case-control exploratory study to compare plasma levels of Ang II and Ang-(1–7) in AD patients vs. age-matched controls. Within each group, we searched for correlations between RAS biomarkers and measures from magnetic resonance imaging (MRI).

**Methods:**

We evaluated patients with AD (n = 14) and aged-matched controls (n = 14). Plasma Ang II and Ang-(1–7) were dosed using ELISA. Brain MRI was performed in a 3 Tesla scan, and a three-dimensional T1-weighted volumetric sequence was obtained. Images were then processed by FreeSurfer to calculate: (1) white matter hypointensities (WMH) volume; (2) volumes of hippocampus, medial temporal cortex, and precuneus. Statistical analyses used non-parametrical tests (Mann-Whitney and Spearman).

**Results:**

Ang-(1–7) levels in plasma were significantly lower in the AD patients than in controls [median (25th–75th percentiles)]: AD [101.5 (62.43–126.4)] vs. controls [209.3 (72–419.1)], *p* = 0.014. There was no significant difference in circulating Ang II. In the AD patients, but not in controls, there was a positive and significant correlation between Ang-(1–7) values and WMH volumes (Spearman’s rho = 0.56, *p* = 0.038). Ang-(1–7) did not correlate with cortical volumes in AD or in controls. Ang II did not correlate with any MRI variable in none of the groups.

**Conclusion:**

If confirmed, our results strengthen the hypothesis that RAS alternative axis is downregulated in AD, and points to a possible interaction between Ang-(1–7) and cerebrovascular lesions in AD.

## Introduction

Worldwide, more than 50 million people suffer from dementia, in 60–70% of the cases caused by Alzheimer’s disease (AD) (World Health Organization (WHO), 2018). The social burden posed by AD places it amid the top priorities for medical research: studies on the subject receive billions of dollars each year in the United States alone (Alzheimer’s association, 2020). Even with the extensive scientific efforts taking place, AD’s pathophysiology remains far from elucidated. Currently, our comprehension of the disease’s mechanisms may be about to face a turning point. So far, most attempts to explain AD’s onset and progression have focused on the brain deposits of beta-amyloid (Aβ) protein. Lately, though, amyloid-targeting drugs have failed to show clinical benefits in successive trials. Such mounting high-quality evidence fuel an active debate around the “amyloid hypothesis” of AD and the limits of its explanatory power (Makin, 2018). Ever more attention is shifting to the disease’s vascular and inflammatory features, encouraging new models to come forward. For instance, one theory suggests that concurrent cerebrovascular dysfunction could prompt AD onset, or synergistically contribute to its progression (Solis et al., 2020). Another hypothesis points to neuroinflammation as a major component of AD’s cognitive decline (Heneka et al., 2015). In this context, the renin-angiotensin system (RAS) emerges as a credible player in AD’s pathogenesis, particularly the RAS’ components involved in cerebrovascular regulation and brain inflammation (Kehoe, 2018).

Primarily remembered as a blood pressure controller, the RAS is in fact a multifaceted system for homeostasis, carrying out diverse and intricate functions. In the past decades, important discoveries transformed the way we think about the RAS. First, active peptides were described and added to the angiotensins’ family, leading to the RAS’ division in two main components: the classical axis, led by its main effector molecule, angiotensin II (Ang II), and the alternative axis, driven by angiotensin (1–7) [Ang-(1–7)]. Second, the concept of “local RAS” (in opposition to systemic RAS) was coined after RAS compounds were found in different organs and tissues, including the central nervous system (CNS) (Mascolo et al., 2017). Following these developments, the RAS has been implicated in medical conditions outside the heart and the kidneys, including neuropsychiatric disorders, AD among them (Rocha et al., 2018). Brain RAS is present to some extent in the hippocampus and other areas affected by AD pathology, where an interaction could take place. More significant, though, are the postulated relations between systemic RAS and AD. At the neurovascular unit level, circulating Ang II deregulates the cerebral blood flow, weakens the blood-brain barrier, and promotes neuroinflammation—all actions that might contribute to AD onset and progression. Plasma Ang-(1–7), on the contrary, might protect against AD-related damages, once it increases cerebral blood flow, reduces blood-brain barrier permeability, and inhibits inflammation. These theoretical perspectives are extensively discussed in our recent review (Ribeiro et al., 2020).

Literature on RAS-AD interaction is profuse and goes beyond theoretical speculation. Experiments with animal models help to build the case for a distinct role for RAS in AD pathogenesis, many even testing RAS as drug target in AD (Saavedra, 2016). Evidence from humans is contrastingly scarce. A recent review found 20 reports of RAS’ molecules measured in AD subjects (Ribeiro et al., 2020). Most of these studies were investigating RAS’ components in the CNS and thus examined brain tissue or cerebrospinal fluid (CSF), with conflicting results. The state of systemic RAS in AD is still largely unexplored. Less than ten studies dosed RAS molecules in AD patients’ blood samples, none of which assessed both RAS axes. More often the focus has been the classical axis, especially the angiotensin-converting enzyme (ACE), and even its role remains unclear. One exception is worth noting: in a case-control study with 228 participants, plasma Ang-(1–7) was significantly lower in the AD group (Jiang et al., 2016a). However, such interesting finding only describes the alternative axis, as other molecules have not been analyzed in the same sample.

Here, we aimed to help shed more light on the complex relationship between AD and systemic RAS. Hence, we have conducted a cross-sectional exploratory study, comparing plasma levels of Ang II and Ang-(1–7) in AD patients vs. cognitively healthy age-matched subjects. Within each group, we searched for correlations between RAS biomarkers and relevant neuroimaging variables, particularly the cortical areas most hit by AD, and markers of cerebrovascular lesions. With our results, we expect to generate hypotheses about the state of both systemic RAS axes in AD, which may help to build inferences about potential mechanisms of interaction.

## Materials and Methods

### Participants

We included 14 patients with mild to moderate AD evaluated at the Neurology Outpatient Clinic of a University Hospital (Hospital das Clínicas da Universidade Federal de Minas Gerais, Belo Horizonte, MG, Brazil). All patients presented with a typical history of progressive episodic memory deficits and showed medial temporal atrophy in brain magnetic resonance imaging (MRI), meeting the AD diagnostic criteria (McKhann et al., 2011). Experienced neurologists and psychiatrists carefully evaluated all patients, to rule out conditions that may mimic AD cognitive impairment. In addition, 10 out of the 14 patients had their CSF analyzed for amyloid beta 42 (Aβ 42), total tau (t-tau) and phosphorylated tau (p-tau). CSF samples were collected by lumbar puncture and biomarkers were measured with a double-sandwich enzyme-linked immunosorbent assay (ELISA) kit (Innogenetics, Gent, Belgium), as previously described (Magalhães et al., 2015). Patients with marked cerebrovascular lesions on brain MRI (Fazekas grade 3) were not included. To further ensure diagnostic accuracy, we followed the participants for at least 24 months after data were collected. In all of them, the disease progressed as expected given the baseline diagnosis.

To compose the control group, 14 older adults without cognitive complaints were recruited within the local community. All participants (AD and controls) underwent a neurological assessment, which included versions of the Mini-Mental State Examination (MMSE) validated in Brazil (Folstein et al., 1975; Brucki et al., 2003). All controls scored 28 or higher in the MMSE. In the clinical interview, participants or family members were asked about time of disease, comorbidities (including hypertension, diabetes, heart failure) and use of medications, especially ACE inhibitors (ACEi) and angiotensin receptor blockers (ARBs). Additional neuropsychological assessment included the Figure Memory Test from the Brief Cognitive Screening Battery (BCSB) for visual episodic memory (Nitrini et al., 2004), the Frontal Assessment Battery (FAB) for executive functions (Beato et al., 2012), and category fluency test (animals in 1 min) for verbal fluency (Machado et al., 2009).

Exclusion criteria for both groups were: (a) history or signs of previous stroke; (b) past neurosurgical procedures; (c) history of other neuropsychiatric conditions, including epilepsy, traumatic brain injury, demyelinating diseases, schizophrenia, bipolar disorder; (d) current or recent (past month) infections; (e) unstable clinical diseases. The Local Research Ethics Committee approved this study’s protocol. All controls and patients (or their legal representatives) were informed about the study and agreed to participate, providing their written informed consent.

### Measurement of Angiotensin Molecules

Peripheral blood was collected from all participants. Blood samples were drawn in vacuum tubes with heparin, centrifuged twice at 1,800 × g for 10 min at 4°C. Plasma samples were then obtained and stored at −70°C until further processing. A quantitative sandwich ELISA was performed to assess plasma levels of Ang-(1–7) (catalog # MBS084052) and Ang II (catalog # MBS028394), following manufacturer’s instructions (MyBioSource, San Diego, CA, United States). Concentrations were measured in pg/ml. The reported sensitivity of the ELISA kits is 2.0 pg/ml for both analytes. All samples were measured in a single assay to avoid inter-assay variability. Our intra-assay variability was lower than 3%. To estimate the balance between RAS alternative and classical axes, the Ang-(1–7)/Ang II ratio was calculated for each subject (Mohite et al., 2018).

### Neuroimages Acquisition and Processing

For all participants, brain MRI was performed in a 3 Tesla Intera-Achieva (Philips, Netherlands) scan. Three-dimensional 1 mm isometric T1-weighted (T1w) volumetric sequence images were acquired with the following parameters: TR: 8.13 ms, TE: 3.71 ms, 256 × 256 matrix, coronal field of view, and slice thickness of 1 mm. Fluid-attenuated inversion recovery (FLAIR) sequence was obtained in all AD subjects (*n* = 14) as well as controls (*n* = 14). FLAIR axial images were evaluated by a neuroradiologist, blinded to subjects’ identity and diagnosis, who classified the deep white matter lesions (WMLs) in the Fazekas’ scale, as described (Fazekas et al., 1987; Kim et al., 2008). Briefly, Fazekas scores are assigned as 0 (absence of WMLs), 1 (punctate WMLs), 2 (early confluent WML), and 3 (large confluent areas of lesion in the white matter).

Before processing, all MRIs were manually assessed for quality control. MRIs with low quality were excluded, e.g., significant presence of motion artifacts, blurring, ringing/truncation, susceptibility phenomenon and bad contrast to noise ratio. On this basis, we excluded from further analysis the MRI data from one of the controls (and none of the AD patients). MRI T1 images were processed for cortical reconstruction and volumetric segmentation using the Freesurfer image analysis suite version 6.0 (FreeSurfer 6.0, 2017). The technical details of these procedures are described in prior publications (Dale and Sereno, 1993; Dale et al., 1999; Fischl et al., 1999a,b, 2001, 2002, 2004a,b; Fischl and Dale, 2000). Cortical areas are anatomically labeled by an automated system (Desikan et al., 2006). Using intensity and continuity information from the entire three-dimensional MR volume, the software processes it (through segmentation and deformation) and calculates cortical thickness, and then cortical volumes. The method has been validated against histological analysis (Rosas et al., 2002) and manual measurements (Han et al., 2006). Following initial automated analysis, manual inspection of the accuracy of post-processing steps was performed. Identifiable errors were corrected through the Freeview visualization tool (from the Freesurfer image analysis tool)^1^. Following manual inspection and any necessary edits, each subject was re-processed through the automated pipeline to account for manual intervention and then manually re-inspected for correction accuracy.

Neuroimaging variables of interest were pre-determined according to their relevance to AD. Hippocampus, entorhinal cortex, and parahippocampal cortex volumes were chosen considering the relevance of medial temporal atrophy for AD (Frisoni et al., 2010). Entorhinal and parahippocampal cortices were combined to compose the medial temporal cortex, as previously defined (de Souza et al., 2012). Precuneus’ cortical volume was also selected given the area’s relevance for disease progression: for instance, this region shows the earliest decline in cerebral perfusion in AD patients (Miners et al., 2016). Finally, the extent of cerebral small vessel disease was assessed using the volume of white matter hypointensities on T1-w images. On T1w sequences, WMLs of presumed vascular origin can appear hypointense, especially when more severe (Wardlaw et al., 2013). Using a probabilistic procedure (Fischl et al., 2002), FreeSurfer differentiates between normally appearing white matter and encompassed white matter signal abnormalities (i.e., hypointensities). The volume of T1w WM hypointensities strongly correlates with distinguished markers of WMLs, such as the Fazekas scale and white matter hyperintensities on T2w and FLAIR sequences (Dadar et al., 2018; Cedres et al., 2020). T1w WM hypointensities may underestimate the true extent of WMLs (Olsson et al., 2013), but have been nonetheless consistently used to measure white matter damage in AD patients and healthy elders (Burns et al., 2005; Salat et al., 2009; Jacobs et al., 2013; Leritz et al., 2014; Fischer et al., 2015; Dadar et al., 2019; Wei et al., 2019; Nemy et al., 2020). All volumes are given in mm^3^ and reported as the sum of left and right hemispheres’ measurements for each individual.

### Statistical Analysis

Statistical analyses were performed using GraphPad Prism 8.0.2 (GraphPad Prism 8.0.2, 2019; GraphPad Software, San Diego, California, United States). To assess normality, we visually inspected the distributions of all continuous variables and run Shapiro-Wilk test. Since data were not normally distributed, non-parametrical tests were used in further analyses. Regarding continuous variables, the two groups (AD and controls) were compared using Mann-Whitney *U*-test. Fisher’s exact test was used to compare categorical (binary) variables among groups. Correlations between variables were calculated using Spearman’s coefficient. Due to the exploratory nature of the study, we have chosen not to adjust for multiple comparisons (Bender and Lange, 2001). When the study sample was divided in three categories, they were compared by Kruskal–Wallis one-way analysis of variance.

## Results

### Clinical Parameters

AD patients and controls were similar in age [(mean ± standard deviation)]: AD (69.5 ± 8.8 years-old) v. controls (66.0 ± 11.0 years-old), *p* = 0.59. As shown in Table 1, AD and control groups were also balanced regarding sex, rates of hypertension and diabetes, and use of ACEi or ARB. Time since first symptoms was, on average, 3.3 years in those with AD (3.3 ± 1.3). As expected, AD patients scored less than controls in MMSE (24.8 ± 2.2 vs. 28.8 ± 0.8, *p* < 0.0001). The results of AD patients were also lower in Figure Memory, categorical fluency (animals) and FAB tests (see Table 1).

**TABLE 1:** AD patients vs. controls: clinical characteristics, plasma angiotensins and neuroimaging.

	AD (*n* = 14)	Controls (*n* = 14)	*p*-value
**Clinical data**			
Age in years—mean ± SD	69.5 ± 8.8	66.0 ± 11.0	0.594^a^
Sex (female)—n (%)	6 (42)	8 (57)	0.706^b^
Hypertension—n (%)	8 (57)	5 (35)	0.449^b^
ACEi or ARB use—n (%)	5 (35)	2 (14)	0.384^b^
Diabetes—n (%)	2 (14)	1 (7)	>0.999^b^
Time of disease (years)—mean ± SD	3.3 ± 1.3	–	–
**CSF biomarkers**			
Aβ 42 (pg/ml)—mean ± SD	572 ± 109*	–	–
t-tau (pg/ml)—mean ± SD	742 ± 271*	–	–
p/tau (pg/ml)—mean ± SD	96 ± 37*	–	–
**Cognitive tests**			
MMSE—median (25th–75th percentile)	24.5 (24–26)	29 (28–30)	**<0.0001**^a^
FMT—5 min Delayed Recall (/10)—median (25th–75th percentile)	4 (2.75–5)	9 (7.75–10)	**<0.0001**^a^
Categorical Fluency (Animals)—median (25th–75th percentile)	12.5 (9.75–14.5)	17.5 (14–19.75)	**<0.001**^a^
FAB—median (25th–75th percentile)	14 (11–15.25)	15.5 (14–17)	**0.035**^a^
**Plasma molecules**			
Ang II pg/ml, median (25th–75th percentile)	61.4 (37.5–88.6)	61.7 (50.3–94.5)	0.602^a^
Ang-(1–7) pg/ml, median (25th–75th percentile)	101.5 (62.4–126.4)	209.3 (72.0–419.1)	**0.014**^a^
Ang-(1–7)/Ang II ratio, median (25th–75th percentile)	1.62 (1.24–2.12)	2.67 (1.63–6.17)	**0.044**^a^
**MRI measures**			
Hippocampus volume^†^ mm^3^ median (25th–75th percentile)	5,523 (5,183–6,504)	7,771 (7,303–8,241)^#^	**<0.0001**^a^
Medial temporal cortex volume **^†^ mm^3^ median (25th–75th percentile)	5,283 (4,322–5,575)	6,858 (6,452–7,258)^#^	**<0.0001**^a^
Precuneus cortical volume^†^, mm^3^ median (25th–75th percentile)	14,454 (13,800–15,029)	16,692 (15,471–17,702)^#^	**0.004**^a^
White matter hypointensities volume, mm^3^ median (25th–75th percentile)	1,912 (1,409–3,436)	1,025 (645–1,697)^#^	**0.007**^a^

*ACEi, Angiotensin-converting enzyme inhibitors; ARB, Angiotensin Receptor Blockers; FAB, Frontal Assessment Battery; FMT, Figure Memory Test; MMSE, Mini Mental State Examination; MRI, Magnetic Resonance Imaging; SD, standard deviation.*

*^*a*^Mann Whitney U-test.*

^*b*^Fisher’s exact test.

*n = 10.

^†^Regions reported as the sum of left and right hemisphere volumes in each subject.

** Medial temporal cortex defined as the combination of entorhinal and parahippocampal cortices.

*^#^ n = 13.*

*Significant findings (*p* < 0.05) are in bold.*

### Angiotensins

**FIGURE 1:**
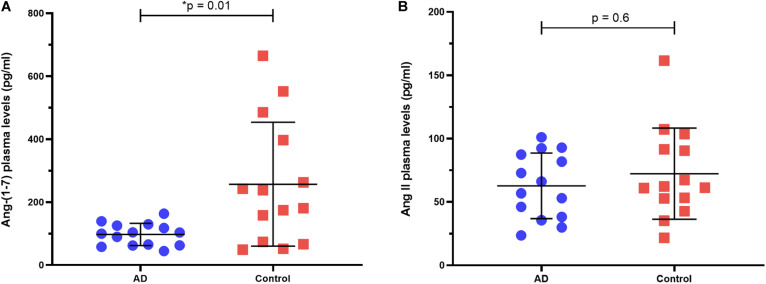
Ang-(1–7) and Ang II plasma levels in AD patients vs. controls. **(A)** AD patients had smaller levels of Ang-(1–7) compared to controls. **(B)** AD patients and controls had similar levels of Ang II. Horizontal bars represent the upper quartile, median, and lower quartile. *P*-values from Mann-Whitney *U*-test.

Ang-(1–7) levels in plasma were significantly lower in the AD patients than in controls [median (25th–75th percentiles)]: AD [101.5 (62.43–126.4)] vs. controls [209.3 (72–419.1)], *p* = 0.014 (Figure 1A). There was no significant difference in circulating Ang II between AD patients [61.45 (37.52–88.6)] and controls [61.7 (50.3–94.5)], *p* = 0.602 (Figure 1B). The difference in Ang-(1–7) levels between groups reflected in the Ang-(1–7)/Ang II ratio, which was significantly lower in AD patients (*p* = 0.044). All results are detailed in Table 1.

To evaluate whether ACEi and ARB use by few patients have influenced our results, we divided the whole sample (AD and controls included) between ACEi/ARB users and non-users. These groups have shown no significant difference in Ang-(1–7) and Ang II levels (see Supplementary Table 1).

### Neuroimaging Variables

MRI variables were first compared between groups. As expected, AD patients presented lower cortical volumes than controls in the hippocampus, medial temporal cortex, and precuneus (all *p* < 0.01). Values are reported in Table 1. Differences in Fazekas grades between the groups were not significant (*p* = 0.21). White matter hypointensities (WMHs) showed a significantly higher volume in AD patients (2,565 ± 1,775 mm^3^) compared to controls (1,204 ± 675 mm^3^), *p* = 0.007.

To verify whether T1w WMHs reflected WMLs in our sample, we grouped all participants (AD and controls) and evaluated the correlation between the Fazekas scale and WMHs volume. Confirming previous findings (Cedres et al., 2020), there was a significant correlation between WMHs and Fazekas grade in our cohort (Spearman’s rho = 0.62, *p* < 0.001). Dividing subjects in three categories according to Fazekas’ grade (0, 1, and 2), we showed WMHs volume was significantly different across three groups (Kruskal-Wallis test, *p* < 0.01). Results of this proof of concept are depicted in Supplementary Figure 1.

### Correlations Between Neuroimaging Variables and Angiotensins

**FIGURE 2:**
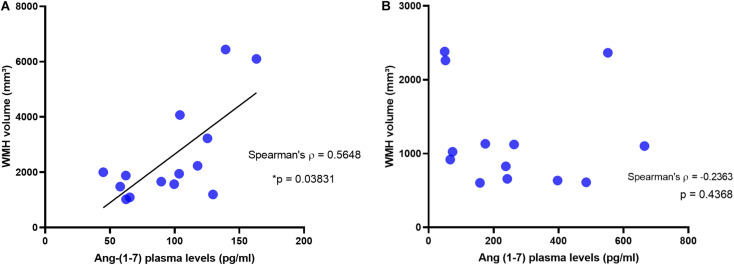
Correlation between White Matter Hypointensities and Plasma Levels of Ang-(1–7) in AD patients and in controls. White matter hypointensities volumes positively correlated with Ang-(1–7) in AD patients **(A)**, but not in controls **(B)**.

In the AD group, no significant correlation was found between plasma Ang II and MRI variables, namely hippocampus, medial temporal cortex, precuneus, and WMHs (see Table 2). In AD patients, Ang-(1–7) levels were not associated with any cortical measure of interest. In contrast, there was a positive and significant correlation between Ang-(1–7) values and WMHs volumes in AD (Spearman’s rho = 0.56, *p* = 0.038). The same relationship was not observed in controls (see Figure 2). In fact, controls did not present any significant correlations between MRI variables and angiotensins. All analyses are detailed in Table 2, whereas the main findings are shown in Figure 2.

**TABLE 2:** Correlations between MRI variables and plasma angiotensins in AD patients and controls.

	Plasma Ang II	Plasma Ang-(1–7)	Ang-(1–7)/Ang II ratio
	
	Spearman’s rho coefficient (95% confidence interval)		
**AD patients**			
Hippocampus volume	−0.05 (−0.57 to 0.5), *p* = 0.868	0.13 (−0.44 to 0.63), *p* = 0.64	0.19 (−0.39 to 0.66), *p* = 0.512
Medial temporal cortex volume	−0.21 (−0.67 to 0.37), *p* = 0.464	0.02 (−0.52 to 0.56), *p* = 0.928	0.26 (−0.32 to 0.7), *p* = 0.357
Precuneus cortical volume	−0.43 (−0.79 to 0.14), *p* = 0.124	−0.21 (−0.67 to 0.37), *p* = 0.463	0.3 (−0.28 to 0.72), *p* = 0.295
White matter hypointensities volume	0.464 (−0.1 to 0.8), *p* = 0.097	**0.56 (0.03 to 0.84),** *p* = **0.038**	−0.05 (−0.58 to 0.49), *p* = 0.844
**Controls**			
Hippocampus volume	0.09 (−0.49 to 0.62), *p* = 0.751	−0.36 (−0.77 to 0.24), *p* = 0.217	−0.38 (−0.77 to 0.22), *p* = 0.196
Medial temporal cortex volume	−0.02 (−0.57 to 0.54), *p* = 0.945	−0.45 (−0.8 to 0.15), *p* = 0.123	−0.36 (−0.77 to 0.24), *p* = 0.214
Precuneus cortical volume	0.22 (−0.39 to 0.69), *p* = 0.47	−0.09 (−0.62 to 0.49), *p* = 0.751	−0.32 (−0.75 to 0.29), *p* = 0.28
White matter hypointensities volume	−0.16 (−0.66 to 0.43), *p* = 0.591	−0.23 (−0.70 to 0.37), *p* = 0.437	−0.4 (−0.78 to 0.21), *p* = 0.176

*Significant findings (*p* < 0.05) are in bold.*

## Discussion

To find whether RAS systemic axes were unbalanced in AD, we compared Ang II and Ang-(1–7) levels between AD patients and cognitively healthy controls. Our results showed that Ang-(1–7) was reduced in AD patients, whereas no difference was found in Ang II levels. Ang-(1–7)/Ang II ratio was lower in AD patients simply reflecting the difference in Ang-(1–7). Then, to investigate if systemic RAS is linked to brain pathology, we looked for correlations between plasma angiotensins and MRI variables. In AD patients, but not in controls, plasma levels of Ang-(1–7) correlated with WMHs. No association was found between angiotensins and selected cortical volumes.

It is noteworthy that all our significant findings regarded the RAS alternative axis’ main peptide, Ang-(1–7). Before our study, Ang-(1–7) was already found to be reduced in AD patients (Jiang et al., 2016a), and mice models (Jiang et al., 2016b). The notion that AD patients may lack Ang-(1–7) is consistent with the peptide’s alleged neuroprotective properties (Farag et al., 2017). To our knowledge, no prior study has evaluated Ang-(1–7) in relation to MRI measurements, neither in AD patients nor in controls. As for Ang II, however, our results contrast with a recent report by Yasar et al. (2020), who found an association between Ang II levels and hippocampal atrophy in cognitively healthy elders. The size of our sample and the dissimilar demographics may account for this difference. Another study (Zhuang et al., 2016) described a higher ACE activity in AD patients compared to controls. This would presumably result in higher Ang II levels, which were not verified in our sample. Differences in the target population may help explain the disparities, as Zhuang and colleagues selected subjects with moderate-to-severe AD.

In our results, it was unsurprising that WMHs’ volume was higher in AD patients. In fact, MRI and post-mortem pathological studies reveal that cerebrovascular lesions are more frequent in AD (Schneider et al., 2004, 2007; Jellinger and Attems, 2005; Attems and Jellinger, 2014; Suemoto et al., 2017; Hase et al., 2018). Classically, AD and cerebrovascular disease are considered independent entities, which often happen together only because the prevalence of both increase with age. Assuming such independence, cerebrovascular lesions would contribute to cognitive decline in AD patients only by reducing the “brain reserve,” thus allowing symptoms to manifest earlier (Kapasi and Schneider, 2016; Raz et al., 2016). This notion has been challenged by mounting evidence of interaction between cerebrovascular disease and AD amyloid and tau pathologies (van Norden et al., 2012; Nucera and Hachinski, 2018; Solis et al., 2020). For instance, cerebral blood flow is dysregulated in AD (Jagust et al., 1997; Roher et al., 2012). Traditionally, ensuing brain hypoperfusion is interpreted as a late consequence of AD pathology, when neurodegeneration diminishes cerebral metabolism, and thus reduces the brain’s need for blood. But there are clinical, radiological and pathological findings suggesting that the mechanisms are likely more complex: control of cerebral perfusion can be disrupted early in AD, and the reduction in blood supply may exceed the decline in metabolic demand (Ruitenberg et al., 2005; Binnewijzend et al., 2014; Hays et al., 2016; Love and Miners, 2016a). Some hypotheses go as far as to state that hypoperfusion can precede (or even induce) other key pathological events in AD (Niedermeyer, 2006; Hays et al., 2016; de la Torre, 2018). Moreover, cerebral blood flow dysregulation is possibly caused by functional changes, rather than a result of structural vascular abnormalities (e.g., atherosclerosis, cerebral amyloid angiopathy) (Kelleher and Soiza, 2013; Love and Miners, 2016b). These changes take place at the level of the neurovascular unit, which adjusts the vascular tone so as that blood supply matches energy demand in the brain (a process named neurovascular coupling). In AD, a malfunctioning neurovascular unit fails to adequately regulate cerebral blood flow and weakens the blood-brain barrier (Benarroch, 2007; Zlokovic, 2011; Iadecola, 2017; Kisler et al., 2017).

If AD pathophysiology is actually influenced by vascular pathology and neurovascular unit dysfunction, then systemic RAS is likely an important player, including its alternative axis (Kangussu et al., 2020). Acting upon the Mas receptor, Ang-(1–7) mediates the alternative axis’ anti-inflammatory, anti-oxidative and vasodilatory properties (Santos et al., 2018). Evidence from preclinical studies suggest that Ang-(1–7) is especially important in brain response to ischemia-hypoxia, increasing cerebral blood flow and preventing blood-brain barrier breakdown (Lu et al., 2008; Zhang et al., 2008; Wu et al., 2015). In animal models of chronic cerebral hypoperfusion, Ang-(1–7) induces tolerance to ischemia and improves cognitive function (Jiang et al., 2014; Xie et al., 2014). Ang-(1–7) has also been studied in mice models of AD. In SAMP8 mice, Ang-(1–7) was reduced and inversely correlated with Tau hyperphosphorylation (Jiang et al., 2016b). When constantly given to SAMP8 mice, Ang-(1–7) counteracted Ang II and prevented cognitive decline (Cao et al., 2019). In Tg2576 mice, upregulating RAS alternative axis reduced amyloid pathology and restored cognition (Evans et al., 2020). It is worth mentioning that no mice model credibly reproduces all the key features of sporadic AD (LaFerla and Green, 2012; Neha et al., 2014). Hence, pathological inferences from animal studies should be interpreted with caution. With such caveat in mind, we can state that preclinical data support the hypothesis of an Ang-(1–7) downregulation contributing to AD.

Against this background, we risk extrapolating our findings to hypothesize that AD patients produce less Ang-(1–7), which may contribute to their disease by diminishing the magnitude of Ang-(1–7) neuroprotective effects. We also speculate that, if confirmed, the positive correlation between plasma levels of Ang-(1–7) and cerebrovascular lesions in AD might result from some sort of response mechanism: for instance, Ang-(1–7) could be upregulated in an effort to counteract the underlying cerebrovascular disease and increase tolerance to ischemia. We assume that such attempt, however, would not raise Ang-(1–7) concentrations to the same levels seen in healthy individuals.

We are aware of this study’s many limitations, starting with the sample size. Besides reducing power and generalizability, having a small sample limited our capacity to adjust results for possible confounders (e.g., hypertension, ACEi or ARB use). To minimize the chance of spurious results, we tried to keep groups balanced concerning variables that might interfere. We still recognize that when few subjects are analyzed, statistical positives can arise only by chance. Regarding the reduced Ang-(1–7) in AD, however, this possibility seems less likely in light of existing data (Jiang et al., 2016a). We also acknowledge that the study would benefit from having a second control group, ideally of patients with another dementia (e.g., vascular dementia). If such third group had been added, it would help to determine whether our findings are specific of AD or common to different dementias. Moreover, one of the inherent disadvantages of the cross-sectional design, not being able to establish causality constrained our attempts to explain mechanistically our main results. Likewise, the lack of histopathological data restricts the consistency of pathophysiological inferences we made. It should also be noticed that in this study, even the biochemical assessment of the RAS pathways was far from complete. To understand why AD patients have lower Ang-(1–7), we would have to look at the protein that produces it, angiotensin-converting enzyme 2 (ACE2). If ACE2 activity was found to be reduced in AD patients, it would explain the immediate mechanism behind their lack of Ang-(1–7). It would also be useful to measure ACE2 concentration together with its activity. Especially if the correlation between ACE2 levels and activity was strong, dosing both would guide future studies about which assays to perform (Chappell, 2015). Despite these limitations, we believe that, for an exploratory study, our methods were appropriate and achieved the goal of generating hypotheses about RAS-AD interaction.

## Conclusion

In conclusion, our study strengthens the hypothesis that RAS alternative axis is downregulated in AD. It also points to a possible interaction between Ang-(1–7) and cerebrovascular lesions in AD patients. We hope these hypotheses will be addressed in the future by larger studies, with longitudinal follow-up and a more comprehensive assessment of the RAS molecules. We believe that, as AD pathogenesis remains largely unelucidated, it is important to follow every lead that may help to explain the disease. If confirmed, our findings corroborate the view that the RAS is a possible player in Alzheimer’s disease pathophysiology.

## Data Availability Statement

The raw data supporting the conclusions of this article will be made available by the authors, without undue reservation.

## Ethics Statement

The studies involving human participants were reviewed and approved by Comitê de Ética em Pesquisa da Universidade Federal de Minas Gerais. The patients/participants provided their written informed consent to participate in this study.

## Author Contributions

VR, AS, and LS designed the study and wrote the protocol. TC proposed the neuroimaging protocol and conducted the MRI analysis. LP and RF planned and conducted the biomarker assays. LS, PC, and AT enrolled participants and performed neurological evaluation. VR undertook the statistical analysis, reviewed by AS and LS. All authors contributed to and have approved the final manuscript.

## Conflict of Interest

The authors declare that the research was conducted in the absence of any commercial or financial relationships that could be construed as a potential conflict of interest.

## Footnotes

1 https://surfer.nmr.mgh.harvard.edu/fswiki/FreeviewGuide

## Supplementary Material

Supplementary Figure 1 Validation of T1-weighted White Matter Hypointensities volume against Fazekas scale in the whole sample. White matter hypointensities volume correlated with Fazekas scale **(A)** and was different across groups defined by Fazekas score **(B)** - *p* = 0.005 (Kruskal-Wallis).

Supplementary Table 1 Ang-(1–7) and Ang II plasma levels in ACEi/ARB users vs. non-users.
