# Supplementary figures and images for: Circulating Angiotensin-(1–7) Is Reduced in Alzheimer’s Disease Patients and Correlates With White Matter Abnormalities: Results From a Pilot Study
Source: Front Neurosci. 2021 Apr 6;15:636754. doi: 10.3389/fnins.2021.636754 (PMC8063113; doi:10.3389/fnins.2021.636754)

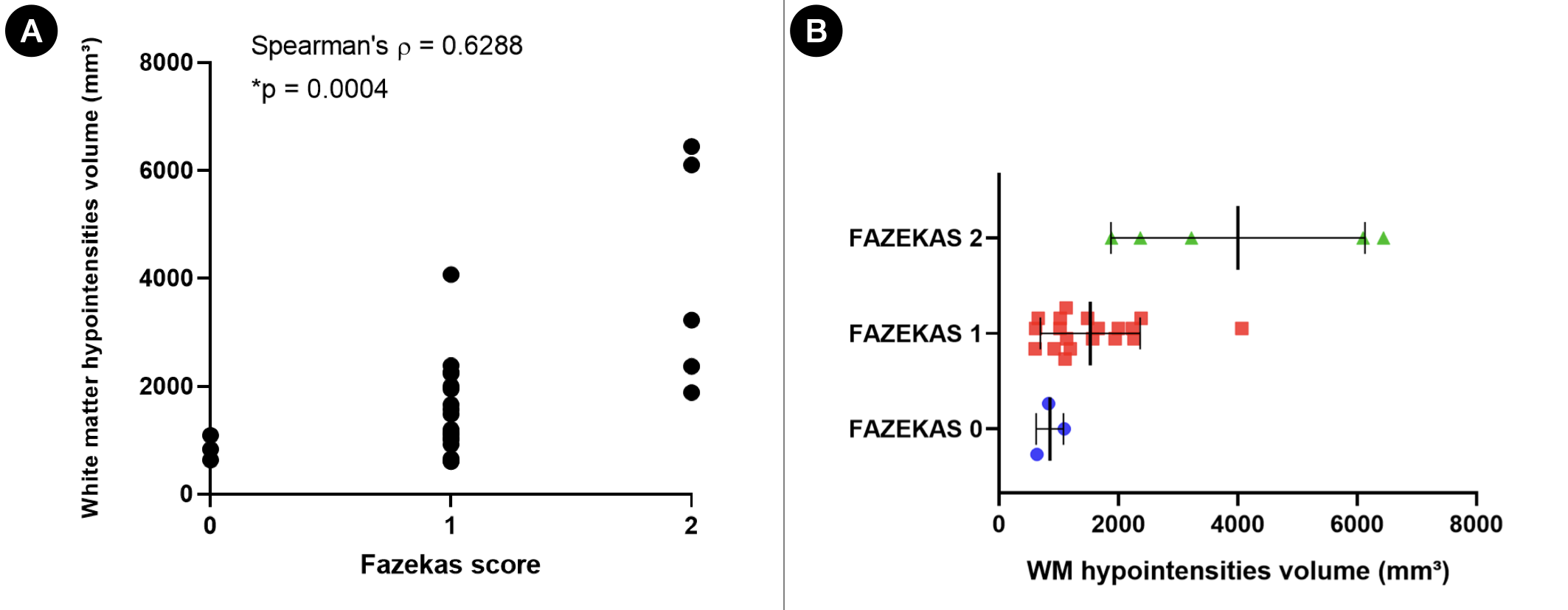

Supplement: Supplementary Figure 1 — Validation of T1-weighted White Matter Hypointensities volume against Fazekas scale in the whole sample. White matter hypointensities volume correlated with Fazekas scale (A) and was different across groups defined by Fazekas score (B) - p = 0.005 (Kruskal-Wallis). [file Image_1.tiff]
