# Supplementary material for: Circulating Angiotensin-(1–7) Is Reduced in Alzheimer’s Disease Patients and Correlates With White Matter Abnormalities: Results From a Pilot Study
Source: Front Neurosci. 2021 Apr 6;15:636754. doi: 10.3389/fnins.2021.636754 (PMC8063113; doi:10.3389/fnins.2021.636754)
Supplement: Supplementary Table 1 — Ang-(1–7) and Ang II plasma levels in ACEi/ARB users vs. non-users. [file Table_1.docx]

**Supplementary Table 1. Ang-(1-7) and Ang II plasma levels in ACEi/ARB users vs. non-users**

|  | ACEi/ARB users (n=7) | ACEi/ARB non-users (n=21) | p-value |
| --- | --- | --- | --- |
| Ang II pg/ml, median (25^th^ - 75^th^ percentile) | 72.77 (42.6 -92.3) | 60.9 (42.1 – 90.9) | 0.603 ^a^ |
| Ang-(1-7) pg/ml, median (25^th^ - 75^th^ percentile) | 104.1 (62.2-129.6) | 139.4 (65.9 – 250.3) | 0.321 ^a^ |
| ACEi: Angiotensin-converting enzyme inhibitors; ARB: Angiotensin Receptor Blockers;  ^a^ Mann Whitney U test; | | | |
